# Current results on the potential health benefits of lutein

**DOI:** 10.17179/excli2016-278

**Published:** 2016-04-28

**Authors:** Jae Kwang Kim, Sang Un Park

**Affiliations:** 1 Division of Life Sciences, College of Life Sciences and Bioengineering, Incheon National University, Incheon, 406-772, Korea; 2 Department of Crop Science, Chungnam National University, 99 Daehak-ro, Yuseong-gu, Daejeon, 305-764, Korea

## ⁯

Dear Editor,

Lutein is a nutritionally beneficial organic tetraterpenoid pigment; its molecular formula and weight are C_40_H_56_O_2 _and 568.87144 g/mol, respectively. It is responsible for the yellow color of fruits and vegetables and is found in high levels in parsley, spinach, kale, egg yolk, and lutein-fortified foods (Shegokar and Mitri, 2012[29]). 

Lutein has a wide range of beneficial health effects including antioxidant, anti-inflammatory, antiatherogenic, antihypertensive, antidiabetic, antiulcer, and anticancer effects (Miyazawa et al., 2013[22]; Johnson, 2014[13]; Erdman et al., 2015[8]; Manayi et al., 2015[19]). Furthermore, it is used to prevent eye diseases including age-related macular degeneration (AMD), cataract, and retinitis pigmentosa (Koushan et al., 2013[15]; Sulich et al., 2015[34]).

The commercial value of lutein is growing with the customary age-related macular degeneration applications. The lutein market is segmented into pharmaceutical, nutraceutical, food, pet foods, cosmetics, and animal and fish feed. Lutein shows a range of biological activities and health benefits in animals; therefore, herein, we have reviewed the most recent studies on lutein and its biological and pharmacological activities (Table 1(Tab. 1); References in Table 1: Song et al., 2015[32]; Sheshappa et al., 2015[30]; Crichton et al., 2015[6]; Lieblein-Boff et al., 2015[17]; Du et al., 2015[7]; Cheng et al., 2015[4]; Qiu et al., 2015[25]; Fatani et al., 2015[9]; Wu et al., 2015[44]; Huang et al., 2015[12]; Li et al., 2015[16]; Han et al., 2015[11]; Rafi et al., 2015[26]; Tian et al., 2015[37]; Casaroli-Marano et al., 2015[2]; Wang et al., 2014[42]; Niesor et al., 2014[23]; Su et al., 2014[33]; Sun et al., 2014[35]; Kon et al., 2014[14]; Promphet et al., 2014[24]; Furlani et al., 2014[10]; Serpeloni et al., 2014[28]; Matsumoto et al., 2014[20]; Vishwanathan et al., 2014[40]; Sen et al., 2014[27]; Min and Min, 2014[21]; Ma et al., 2014[18]; Xu et al., 2013[45]; Binawade et al., 2013[1]; Chang et al., 2013[3]; Tian et al., 2013[38]; Yajima et al., 2013[47]; Sung et al., 2013[36]; Costa et al., 2013[5]; Wang et al., 2013[41]; Yao et al., 2013[48]; Xu et al., 2013[46]; Vijayapadma et al., 2014[39]; Woo et al., 2013[43]; Sindhu et al., 2012[31]).

| **Key findings** | **Reference** |
|---|---|
| Interestingly, the combination of lutein with vitamin C which is responsible to improve the total antioxidant defense system properties in Sprague-Dawley (SD) rats is found by Song et al. (2015) | Song et al., 2015 |
| The intestinal absorption of both micellar and dietary lutein has been enhanced by phosphatidylcholine content. This implies that lutein deficiency in aged rats can be improved by phosphatidylcholine. On the other hand lutein bioavailability also enriched in lutein deficiency rats by phosphatidylcholine which is either in free or bound form with food matrices. | Sheshappa et al., 2015 |

EXCLI Journal 2016;15:308-314 – ISSN 1611-2156
Received: March 25, 2016, accepted: April 23, 2016, published: April 28, 2016

| Key findings | Reference |
|---|---|
| Higher intakes of lutein-containing foods, including green leafy vegetables, were associated with higher levels of physical activity. Increasing the consumption of lutein-rich foods may have a positive effect on levels of physical activity. | Crichton et al., 2015 |
| In order to identify the role of lutein in human brain, study of relationship between lutein and metabolic pathways is an important and useful criterion. Fundamental understanding of the relationships between lutein correlation with lipid pathway metabolites, energy pathway metabolites, brain osmolytes, amino acid neurotransmitters, and the antioxidant homocarnosine plays an important role for further analysis specifically in brain development. | Lieblein-Boff et al., 2015 |
| Lutein treatment exerted potent antioxidant and anti-inflammatory effects and offered significant cytoprotection against alcohol-induced liver injury. | Du et al., 2015 |
| Lutein protects against skeletal ischemia/reperfusion injury by downregulating oxidative stress and inflammatory mechanisms. | Cheng et al., 2015 |
| Lutein has positive effects on non-alcoholic fatty liver disease via the modulation of hepatic lipid accumulation and insulin resistance. | Qiu et al., 2015 |
| Lutein has potential beneficial effects in diabetes-induced testicular damage, probably through its antioxidant and anti-inflammatory properties. | Fatani et al., 2015 |
| Lutein attenuates neuroinflammation in lipopolysaccharide-activated BV-2 microglia, partly through the inhibition of p38-, c-Jun N-terminal kinase (JNK)-, and Akt-stimulated NF-κB activation and promotion of extracellular signal-regulated kinase-induced NF-E2-related factor 2 activation, suggesting that it has great potential as a nutritional preventive strategy in inflammation-related neurodegenerative disorders. | Wu et al., 2015 |
| Long-term lutein supplementation could increase serum lutein concentration, macular pigment optical density (MPOD), and visual sensitivity in early age-related macular degeneration (AMD) patients. The advisable long-term lutein dosage for early AMD treatment is 10 mg daily. | Huang et al., 2015 |
| Lutein has antioxidant properties and can thus prevent hepatotoxicity. This finding also suggests that dietary lutein may be a potential treatment for liver diseases, especially arsenicosis. | Li et al., 2015 |
| New evidence supports the antiatherogenic properties of lutein and the mechanism of action underlying its atherosclerosis-preventive effect involves improvements in oxidative stress and lipid metabolism. | Han et al., 2015 |
| Lutein modulates the expression of growth- and survival-associated genes in prostate cancer cells. | Rafi et al., 2015 |
| In the case of lutein supplementation it inhibits the systemic activation of the complement system. This finding is the strong evidence for the reported beneficial effects about the management of AMD. | Tian et al., 2015 |
| It is important to mention here that lutein/zeaxanthin-based dye solutions accomplished a safe profile for *in vitro* and *in vivo* studies. This result has been shown as a better choice for staining intraocular structures. | Casaroli-Marano et al., 2015 |
| Even though lutein supplementation is useful for enhancing visual acuity based on improving MPOD done by Wang et al. (2014), but still more research work is still needed to give appropriate conclusive evidence. In the case of dosage per day analysis it is found that milder dose is much effective compared to higher dose. | Wang et al., 2014 |
| The intestinal absorption of lutein and zeaxanthin may be improved by physiological and pharmacological interventions affecting high-density lipoprotein (HDL) metabolism. | Niesor et al., 2014 |

EXCLI Journal 2016;15:308-314 – ISSN 1611-2156
Received: March 25, 2016, accepted: April 23, 2016, published: April 28, 2016

| Key findings | Reference |
|---|---|
| Lutein can inhibit platelet-derived growth factor-BB induced migration of retinal pigment epithelium cells through the inhibition of both cytoplasmic and mitochondrial Akt activation. | Su et al., 2014 |
| It is well known that lutein has antioxidant properties. That administration of lutein affords strong neuroprotective effect against transient cerebral ischemic injury is the strong evidence for its exclusive antioxidant properties. | Sun et al., 2014 |
| Lutein-fortified formula was safe and well-tolerated and supported the physical growth of evaluated infants. | Kon et al., 2014 |
| Silk lutein extract enhanced both innate and adaptive immune functions, and may prove to be an effective supplement to strengthen immunity. | Promphet et al., 2014 |
| Lutein and zeaxanthin alone or in association with brilliant blue showed a good safety profile in this experimental model. | Furlani et al., 2014 |
| Lutein might scavenge reactive species and induce the expression of genes related to a better antioxidant response, thereby improving oxygen transport. The improvement in the redox state of cells through lutein treatment could be related to the antigenotoxic and antioxidant effects of lutein. | Serpeloni et al., 2014 |
| It is important to mention here that the voluntary running of rats is the very good choice to enhance effective utilization of lipids. Interestingly the lutein level in the blood of rats significantly decreased with exercise. Therefore voluntary running and lutein fortified milk both are the combined source for utilization of lipids. | Matsumoto et al., 2014 |
| Lutein is a promising content in medical field especially neural development in preterm infants. Regarding the preferential accumulation and maintenance of lutein have been studied and reported by Vishwanathan et al. (2014). This study is a pathway for further investigations regarding preterm infants. | Vishwanathan et al., 2014 |
| Higher lutein, zeaxanthin, and vitamin C concentrations in plasma are incorporated with prolonged leukocyte telomere length in common elderly humans and indicate a defensive role of these vitamins in telomere maintenance. | Sen et al., 2014 |
| Nowadays Alzheimer's disease (AD) mortality in adults becomes global concern. Lycopene, lutein and zeaxanthin are the important factors in serum which are responsible to induce AD mortality in adults. From the results it is evident that above mentioned three factors should be high in serum level. Therefore it is concluded that from the findings high intake of lycopene, lutein and zeaxanthin enriched foods only can meet out the requirements to reduce the risk of AD mortality. | Min and Min, 2014 |
| Nowadays age-related cataract (ARC) has paramount of importance among aged people. Lutein and zeaxanthin contents are suitable candidates to reduce the risk of age-related cataract (ARC). Hence the proper intake of lutein and zeaxanthin plays key source for ARC prevention especially for nuclear cataract. | Ma et al., 2014 |
| The novel powerful agents named lutein and zeaxanthin which are having great importance in AMD therapy successfully extracted from *Fructus lycii*. *In vitro* and *in vivo* studies clearly explains beneficial role of active components lutein and zeaxanthin in AMD therapy. | Xu et al., 2013a |
| Lutein is a promising material extensively used for body weight and neurobehavioral alterations, attenuated oxidative stress, and improved the activity of the mitochondrial enzymes complex in rat brain. The performance of lutein against the Huntington's disease is also studied and reported. | Binawade et al., 2013 |

| Key findings | Reference |
|---|---|
| Intestinal damage during conventional chemotherapy is a major challenge in rats for researches. Interestingly, lutein pre-treatment is a best choice to prevent intestinal damage. Form this study the methotrexate-induced apoptosis of IEC-6 cells originating from rat jejunum crypt was studied and explained in proper way. | Chang et al., 2013 |
| In order to control the inflammatory pathway of the innate immune system lutein is an appropriate choice for researches. It possesses simple method to control the circulating levels of complement factors includes C5a and C3d. | Tian et al., 2013 |
| Constant light conditions during the perinatal period may induce some neuronal abnormalities in both offspring and mother that may be partially ameliorated by the antioxidant effects of dietary lutein. | Yajima et al., 2013 |
| Lutein has significant antihypertensive and antioxidant effects against hypertension induced by N(G)-nitro-L-arginine methyl ester hydrochloride in rats. | Sung et al., 2013 |
| Usage of lutein has been extensively applied in medical field especially for preterm infants due to its excellent biological antioxidant property. Many researches are going on to understand the excellent biological activity of lutein for preterm infants. But this research area is still required novel and innovative pathways and solutions in developing new drugs for preterm infants. | Costa et al., 2013 |
| Lutein supplementation reduced the levels of biomarkers of cardiovascular diseases by decreasing the lipid peroxidation and inflammatory response by increasing plasma lutein concentration and antioxidant capacity. | Wang et al., 2013 |
| Daily supplementation with 20 mg of lutein increases macular pigment optical density. Lutein may improve the ability to drive at night and to perform other spatial discrimination tasks under low illumination. | Yao et al., 2013 |
| Atherosclerosis is a well-known vascular disease. It is reported that high lutein level in serum can regulate serum lipids and cytokines which are responsible for early atherosclerosis. From this study it is evident that lutein level in serum plays a key role to control vascular diseases. | Xu et al., 2013b |
| Lutein has protective effects against B(a)P-induced oxidative stress, possibly by combating oxidative stress by its free radical scavenging activity. | Vijayapadma et al., 2014 |
| Fundamental understanding of lutein exhibits rich and fascinating properties of lutein in biomedical field. It is a potent neuroprotective agent that can salvage photoreceptors in rats. From the observations it is concluded that lutein is a milestone of drug designing process for various diseases. | Woo et al., 2013 |
| The antiulcer activity of lutein may be attributable to the inhibition of oxidative stress produced by alcohol. These findings suggest the potential therapeutic use of lutein as an effective antiulcer agent. | Sindhu et al., 2012 |

***Acknowledgements***

***Conflict of interest***

The authors declare no conflict of interest.

## Acknowledgements

This research was supported by Golden Seed Project funded by Ministry of Agriculture, Food and Rural Affairs (MAFRA), Ministry of Oceans and Fisheries (MOF), Rural Development Administration (RDA) and Korea Forest Service (KFS).

## Conflict of interest

The authors declare no conflict of interest.

## Figures and Tables

**Table 1:**
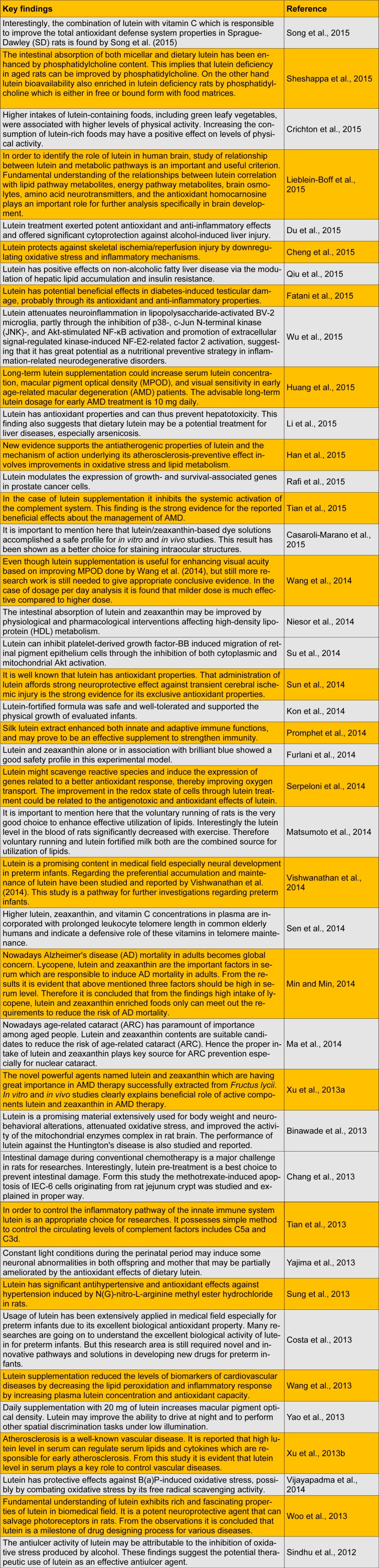
Recent studies on lutein and its biological and pharmacological activities
